# Antibodies Targeting the Cell Wall Induce Protection against Virulent Mycobacterium bovis Infection

**DOI:** 10.1128/spectrum.03431-22

**Published:** 2023-02-27

**Authors:** Mengjin Qu, Zhengmin Liang, Yulan Chen, Yuanzhi Wang, Haoran Wang, Ziyi Liu, Yiduo Liu, Yuhui Dong, Xin Ge, Hao Li, Xiangmei Zhou

**Affiliations:** a College of Veterinary Medicine, China Agricultural University, Beijing, China; Wuhan University

**Keywords:** BCG, BCR repertoire, *Mycobacterium bovis*, antibody, cell wall

## Abstract

Accumulating evidence indicates that antibodies can protect against some intracellular pathogens. Mycobacterium bovis is an intracellular bacterium, and its cell wall (CW) is essential for its virulence and survival. However, the questions of whether antibodies play a protective role in immunity against M. bovis infection and what effects antibodies specific to the CW of M. bovis have still remain unclear. Here, we report that antibodies targeting the CW of an isolated pathogenic M. bovis strain and that of an attenuated bacillus Calmette-Guérin (BCG) strain could induce protection against virulent M. bovis infection *in vitro* and *in vivo*. Further research found that the antibody-induced protection was mainly achieved by promoting Fc gamma receptor (FcγR)-mediated phagocytosis, inhibiting bacterial intracellular growth, and enhancing the fusion of phagosomes and lysosomes, and it also depended on T cells for its efficacy. Additionally, we analyzed and characterized the B-cell receptor (BCR) repertoires of CW-immunized mice via next-generation sequencing. CW immunization stimulated BCR changes in the complementarity determining region 3 (CDR3) isotype distribution, gene usage, and somatic hypermutation. Overall, our study validates the idea that antibodies targeting the CW induce protection against virulent M. bovis infection. This study highlights the importance of antibodies targeting the CW in the defense against tuberculosis.

**IMPORTANCE** M. bovis is the causative agent of animal tuberculosis (TB) and human TB. Research on M. bovis is of great public health significance. Currently, TB vaccines are mainly aimed at eliciting protection by enhancement of cell-mediated immunity, and there are few studies on protective antibodies. This is the first report of protective antibodies against M. bovis infection, and the antibodies had both preventive and even therapeutic effects in an M. bovis infection mouse model. Additionally, we reveal the relationship between CDR3 gene diversity and the immune characteristics of the antibodies. These results will provide valuable advice for the rational development of TB vaccines.

## INTRODUCTION

Mycobacterium bovis, a robust and tricky intracellular bacterium, infects cattle, domestic and wild animals, and even humans (1). The World Health Organization (WHO) reported that an estimated 140,000 new tuberculosis (TB) cases and 11,400 deaths were caused by M. bovis globally in 2019 (2). Bacillus Calmette-Guérin (BCG), an attenuated strain of pathogenic M. bovis, remains the only TB vaccine approved for clinical use since 1921. Although the protective effect of BCG against TB is limited, recent studies showed that intravenous vaccination with BCG could provide substantial resistance to Mycobacterium tuberculosis infection in nonhuman primates (3). Additionally, compared to traditional intradermal delivery of BCG, intravenous BCG immunization induced robust antibody responses, and IgM in the plasma and lungs has been proved to be strongly associated with bacterial burden reduction (3, 4).

Accumulating evidence indicates that antibodies (Abs) play a protective role in M. tuberculosis infection (5–11). Abs targeting the M. tuberculosis surface isolated from health care workers exposed to M. tuberculosis were protective against M. tuberculosis infection in a mouse infection model (7). Abs against the arabinomannan (AM) of the M. tuberculosis cell wall (CW) derived from asymptomatic subjects in TB-endemic areas could also defend against M. tuberculosis infection (8). Moreover, Abs against the M. tuberculosis surface-exposed phosphate transporter subunit PstS1 isolated from patients with active TB infection reduced the lung bacterial load of M. tuberculosis-infected mice by 50% (9). These results show strong evidence that Abs against specific M. tuberculosis surface antigens can protect against M. tuberculosis infection.

The CW of mycobacteria is composed mainly of a complex array of lipids and carbohydrates that provide an impermeable fortress against hydrophilic drugs, and it is essential for their virulence and survival (12, 13). Mycolylarabinogalactan-peptidoglycan complex (mAGP), the core component of the CW, is a crucially important virulence factor for M. tuberculosis, and a variety of drugs, such as ethambutol, isoniazid, and ethionamide, successfully target the synthetic and assembly pathways of various components of mAGP (12–15). Additionally, both the plasma membrane and periplasm of the CW possess glycophospholipids, such as phosphatidyl-myo-inositol mannosides (PIMs), lipomannan (LM), and lipoarabinomannan (LAM), which have critical roles in modulating the host immune response during infection (12, 13). BCG-CW, as an ingredient of complete Freund’s adjuvant, can enhance the protective efficacy of vaccine by enhancing Th1 and Th17 immune responses and inducing dendritic cell (DC) maturation (16–18). However, the protective effect of Abs elicited by the BCG-CW has not been evaluated, and the functional characterization of its protective Abs is still lacking.

In this study, we explored the protection conveyed by BCG-CW-induced Abs and analyzed the B-cell receptor (BCR) repertoires evoked by BCG-CW using next-generation sequencing. Meanwhile, the CW of strain C68004 was selected as a reference. C68004 is a highly virulent strain of M. bovis isolated from a dairy cow with bovine TB in Beijing in 1952 and widely used in the study of M. bovis (19–22). Our results show that the CW induces a robust antibody response in mice, and these Abs, especially those induced by C68004-CW, can inhibit the growth of M. bovis *in vivo* and *in vitro*. Furthermore, CW immunization results in different characteristics of BCRs in terms of the complementarity determining region 3 (CDR3) isotype distribution, gene usage, and somatic hypermutation (SHM). Our study demonstrates that Abs targeting the CW can induce protection against M. bovis infection and facilitates the understanding of CW antigen-driven B-cell responses, which may provide new ideas for developing novel TB vaccines.

## RESULTS

### Protective efficacy of the CW in mice.

**FIG 1:**
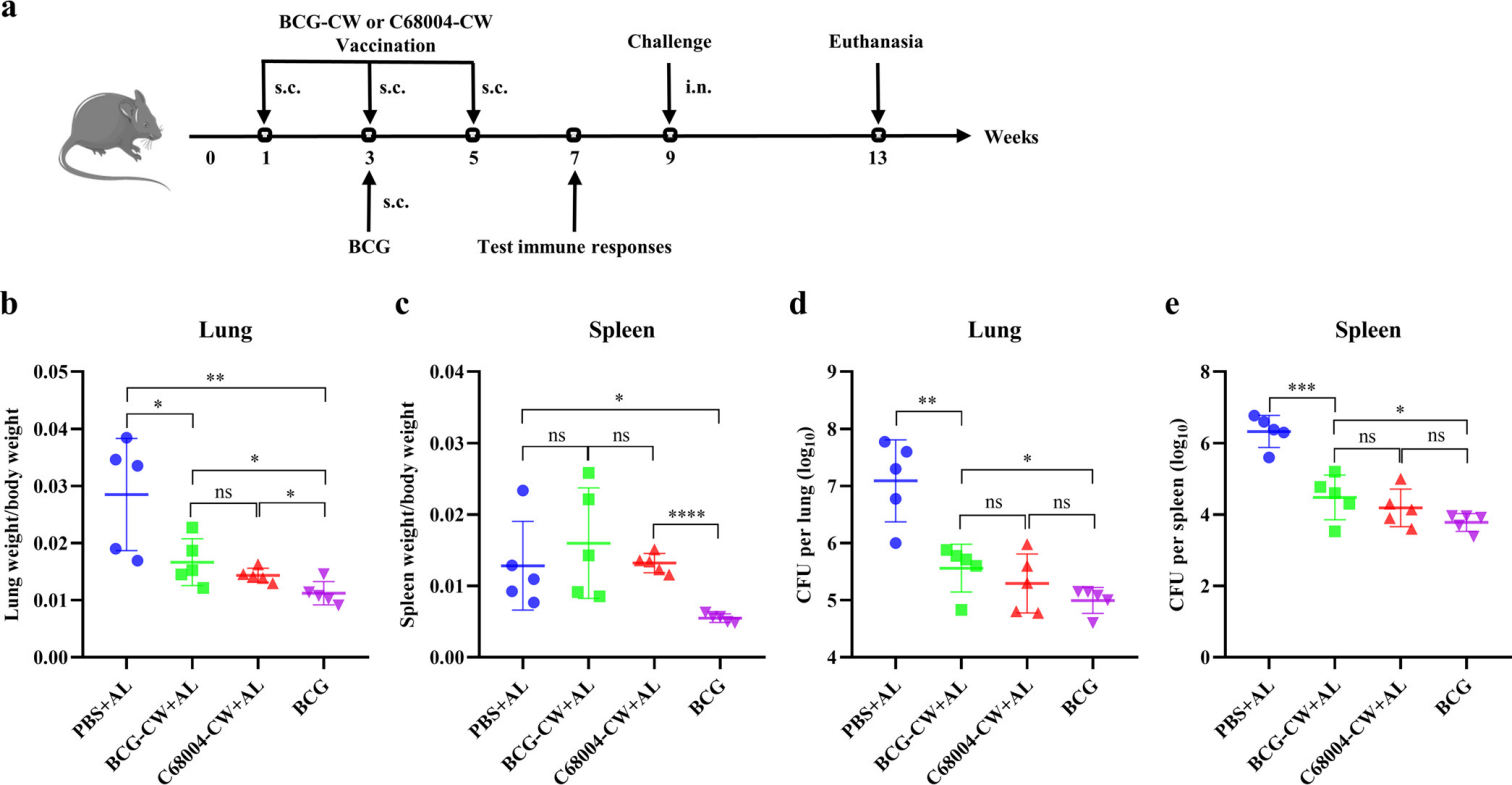
Immunization with BCG-CW or C68004-CW protects against M. bovis infection. (a) Schematic model of the mouse protection assay. C57BL/6 mice were randomly divided into four groups (*n *= 8 per group). Mice in the two groups were inoculated subcutaneously (s.c.) 3 times with 50 μg of BCG-CW or C68004-CW, including 1% (wt/vol) alum, at 2-week intervals. Negative-control mice received 3 s.c. injections of PBS, including 1% (wt/vol) alum, while BCG control mice were vaccinated s.c. once with 1 million BCG. Two weeks after the last immunization, the immune responses of the mice (*n *= 3 per group) were measured. Immunized mice (*n *= 5 per group) were infected with 100 CFU of M. bovis (C68004 strain) via the intranasal (i.n.) route 4 weeks after the last immunization and sacrificed 4 weeks after challenge for organ lesion scores and bacterial enumeration. (b to e) The organ coefficients (ratio of organ weight to the total body weight) of the lung (b) and spleen (c) and the numbers of viable bacteria in the lungs (d) and spleen (e) were determined. Data are shown as mean values ± standard deviations (SD) and were analyzed with the unpaired two-tailed *t* test. *, *P < *0.05; **, *P < *0.01; ***, *P < *0.001; ns, not significant.

To investigate the protection induced by the CW, C57BL/6 mice were immunized 3 times with purified BCG-CW or C68004-CW isolated from bacteria grown in a detergent-free medium (Fig. S1 in the supplemental material) and then challenged with M. bovis and euthanized 4 weeks later to assess the bacterial loads in lungs and spleens (Fig. 1a). Moreover, the safety of CW vaccination was first monitored before the challenge to exclude the influence of CW autotoxicity. Our data revealed that CW immunization via the subcutaneous (s.c.) route had no toxic effects on important internal organs and did not affect body weight gain in mice for a certain period of time (Fig. S2a and b). After the challenge, the body weight of CW-immunized mice remained as relatively stable as that of BCG-vaccinated mice (Fig. S2b). At 4 weeks after the challenge, the lungs of CW-immunized mice presented a lower organ coefficient (ratio of organ weight to total body weight) and fewer and milder nodular lesions than the lungs of the PBS group (Fig. 1b and Fig. S2c). These results indicated that CW immunization could alleviate the symptoms of M. bovis infection in mice. Surprisingly, the spleens of some BCG-CW and C68004-CW-immunized mice were larger than those of the PBS group (Fig. 1c and Fig. S2c). The enlarged spleens may be due to a robust immune response triggered by CW. BCG-CW- and C68004-CW-immunized mice had significantly lower bacterial loads in lungs and spleens than the PBS group, and a statistical difference was not observed in the bacterial loads of the C68004-CW group and the BCG group (Fig. 1d and e). Although no statistical difference was observed between the BCG-CW group and the C68004-CW group, the average bacterial load of the BCG-CW group was higher than that of the C68004-CW group (Fig. 1d and e), which showed that the protective effect induced by C68004-CW was more excellent than that induced by BCG-CW. In conclusion, immunization with BCG-CW or C68004-CW could provide protection against M. bovis infection in the mouse model.

### Antibody responses to CW in mice.

**FIG 2:**
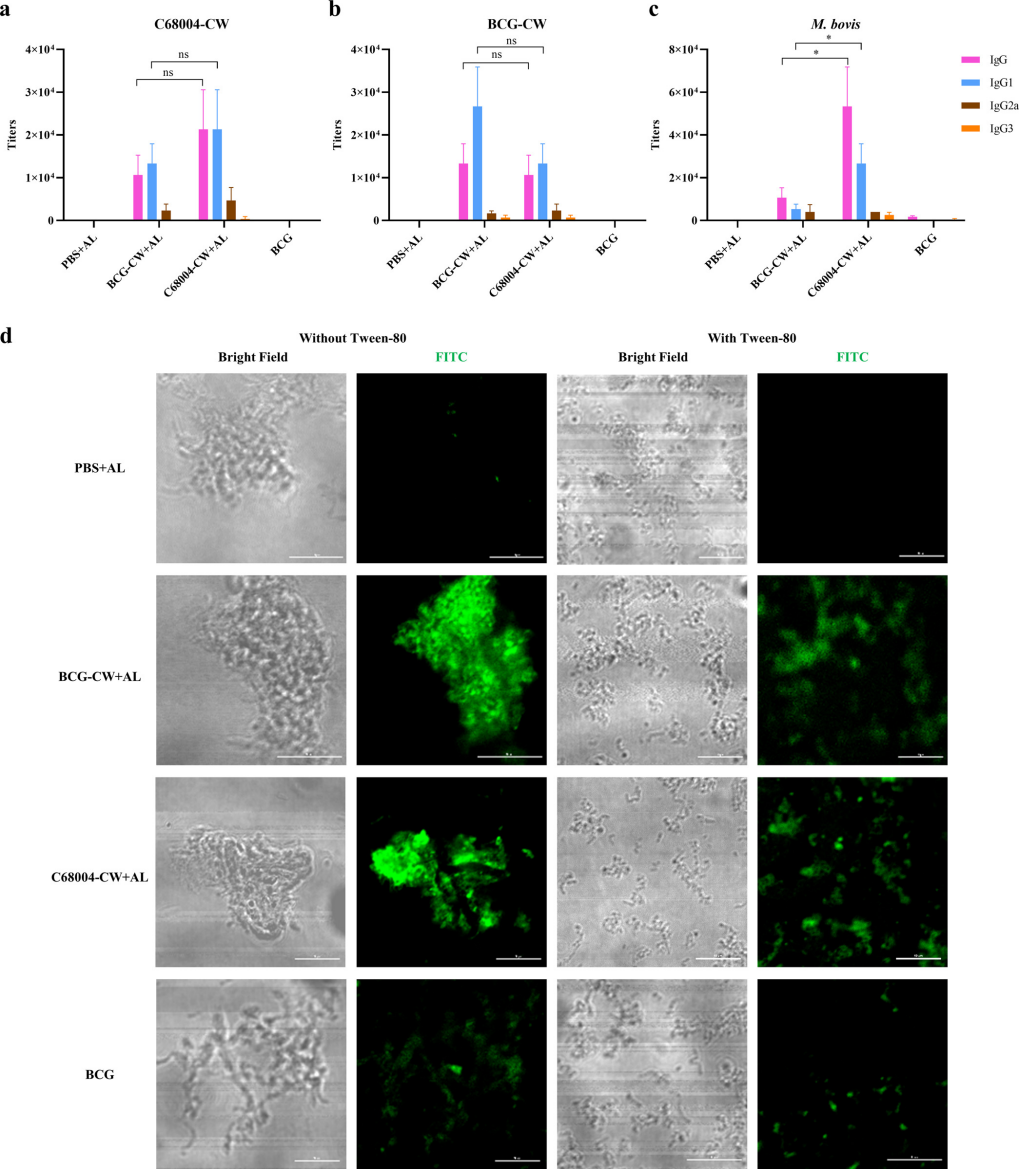
Antibody responses to CW immunization in mice. Two weeks after the mice were last immunized, the sera of immunized mice (*n *= 3 per group) were collected to test for antibody responses. (a to c) The levels of serum antibody titers were determined using ELISA against C68004-CW (a), BCG-CW (b), and M. bovis (c). Data are shown as mean values ± SD and were analyzed with the unpaired two-tailed *t* test. *, *P < *0.05; ns, not significant. All experiments were repeated at least 3 times. (d) Immunofluorescence microscopy of M. bovis (C68004 strain) cultured with and without Tween 80 and incubated with the immunized mouse sera, including alum (+AL) where indicated. The brightness and contrast of the images were adjusted with the identical parameter settings by using ImageJ software. Scale bar: 10 μm.

To test the immunological response of CW fractions, serum antibody levels were determined 2 weeks after the last immunization (Fig. 1a). BCG-CW and C68004-CW induced high levels of specific IgG, mainly IgG1 (Fig. 2a and b). The BCG-CW- and C68004-CW-binding antibody responses were very similar in their diversity (Fig. 2a and b), indicating that the two antigens had a cross-reactivity. The antibody levels induced by BCG immunization were almost comparable to those of the phosphate-buffered saline (PBS) group, corresponding to previously reported results (23). Furthermore, the reactions of the serum with whole bacterial cells were measured (Fig. 2c). The levels of M. bovis-specific IgG and IgG1 induced by C68004-CW were significantly higher than those induced by BCG-CW, which might be because the coated antigen in the plate was the strain C68004, making C68004-CW serum have a higher affinity.

To further determine whether the CW immunization serum could recognize bacterial surfaces, immunofluorescence experiments were performed (Fig. 2d). Tween 80 is known to be a mild detergent that can strip away part of the mycobacterial capsule components (24). BCG-CW and C68004-CW serum bound to bacteria with or without Tween 80 treatment, but unrelated serum did not. Importantly, the binding of BCG-CW and C68004-CW serum to Tween 80-treated bacteria was significantly weaker, indicating that most of the materials identified by the serum were present on the capsule of bacteria.

Collectively, these results demonstrate that immunization with BCG-CW and C68004-CW induces potent and specific antibody responses that are primarily directed against mycobacterial surfaces, especially capsules.

### Opsonization of CW serum *in vitro*.

**FIG 3:**
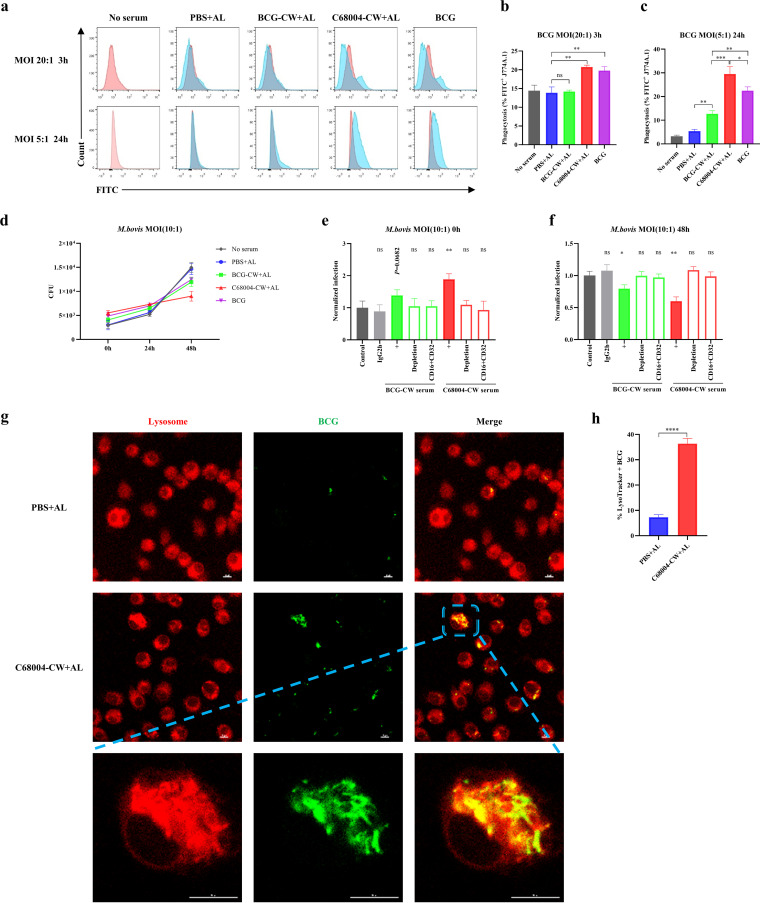
CW serum inhibits M. bovis infection *in vitro*. (a) J774A.1 cells were infected with FITC-conjugated BCG with an MOI of 20:1 or 5:1 and coincubated with 10% heat-inactivated serum for 3 h or 24 h. The histograms show the frequencies of intracellular FITC-conjugated BCG. For each serum, the binding histogram is shown in blue, and the blank control histogram is in red. (b and c) Quantification of the data from the experiment whose results are shown in panel a. (d) J774A.1 cells were incubated with heat-inactivated serum and infected with M. bovis (MOI of 10) for 3 h. Then, the cells were washed and resupplemented with heat-inactivated serum, and this time point was counted as 0 h postinfection. The cells were lysed and harvested for CFU counting at 0 h, 24 h, and 48 h postinfection. The slope of the line segment indicates the growth rate of the bacteria. (e and f) The opsonization by BCG-CW serum and C68004-CW serum following anti-CW Ab depletion or CD16/CD32 blocking was evaluated at 0 h (e) and 48 h (f) postinfection in J774A.1 cells infected with M. bovis (MOI of 10). IgG2 was the isotype control of anti-CD16/CD32 monoclonal Ab. The data in each group were compared to the average CFU infection in the control group, which was normalized to 1. (g) Confocal microscopy was used to analyze the colocalization of endogenous BCG/phagosomes (green) with lysosomes (red) in J774A.1 cells after infection with FITC-conjugated BCG at an MOI of 20 for 1 h, followed by coincubation with PBS or C68004-CW immunization serum. Scale bar, 10 μm. (h) Quantification of the percentages of colocalization of BCG/phagosomes and lysosomes in the experiment whose results are shown in panel g. At least 250 cells in 10 fields were counted in each group. Data are shown as mean values ± SD and were analyzed with the unpaired two-tailed *t* test. *, *P < *0.05; **, *P < *0.01; ***, *P < *0.001; ****, *P < *0.0001; ns, not significant. All experiments were repeated at least 3 times.

The sera from immunized mice were further used to determine the role of humoral immunity in protection against M. bovis infection *in vitro*. First, to investigate the effect of CW serum on bacterial entry, J774A.1 macrophages were coincubated with fluorescein isothiocyanate (FITC)-conjugated BCG and heat-inactivated immunized-mouse serum. After 3 h of incubation with a multiplicity of infection (MOI) of 20, although BCG-CW serum did not enhance the phagocytosis of BCG by macrophages compared with the phagocytic effect of serum from the control group, C68004-CW serum showed a significantly enhanced effect (Fig. 3a and b). After 24 h of incubation with an MOI of 5, BCG-CW serum significantly enhanced the uptake of bacteria by phagocytes, while the phagocytic effect of C68004-CW serum was further amplified (Fig. 3a and c). These results suggested that C68004-CW serum had more potential than BCG-CW serum to enhance BCG macrophage phagocytosis in both high-dose and low-dose M. bovis infection. Second, to explore the effect of CW serum on the intracellular growth of M. bovis, J774A.1 cells were infected with M. bovis, coincubated with heat-inactivated serum, and harvested for enumeration of viable bacteria. We found that the growth rate of M. bovis in J774A.1 macrophages was obviously reduced in the presence of CW immunization serum, especially C68004-CW serum (Fig. 3d). Importantly, these functions of enhanced phagocytosis and inhibition of intracellular bacterial growth disappeared after depletion of anti-CW Abs (Fig. 3e and f), suggesting that these phagocytic effects were due to anti-CW Abs. Of note, CW serum did not immediately inhibit infection, but it increased bacterial clumps in macrophages postinfection, suggesting that CW serum performed antibody-dependent opsonization of bacteria through Fc gamma receptors (FcγRs). To further assess the contribution of FcγR effectors, we selectively blocked the major FcγRs expressed on macrophages, CD16 (FcγRIII) and CD32 (FcγRII). The results indicated that blocking CD16 and CD32 together abolished the phagocytic and inhibitory activities of CW serum (Fig. 3e and f). These results suggested that anti-CW Abs opsonized M. bovis and make it more susceptible to phagocytosis, which was mediated by FcγRs. Furthermore, murine BCG serum, as reported for human BCG serum (10), opsonized M. bovis, which was manifested as enhancing the phagocytic ability of macrophages against M. bovis and inhibiting the growth of M. bovis (Fig. 3a and d).

Previous studies have shown that Abs specific to M. tuberculosis can increase phagosome-lysosome (P-L) fusion, thereby limiting the growth of M. tuberculosis (6, 8, 10, 25). Therefore, we next examined whether CW serum could affect P-L fusion. C68004-CW serum was selected as the representative serum based on the highest increase of BCG phagocytosis and was incubated with BCG and J774A.1 macrophages. As expected, the percentage of P-L fusion was considerably higher after the treatment with C68004-CW serum than after treatment with control serum (Fig. 3g and h), suggesting that C68004-CW serum could enhance the P-L fusion.

Altogether, our results suggest that anti-CW Abs, especially anti-C68004-CW Abs, can enhance FcγR-mediated M. bovis phagocytosis, restrict the intracellular growth of M. bovis, and increase P-L fusion in J774A.1 macrophages.

### CW serum inhibits M. bovis in mice.

**FIG 4:**
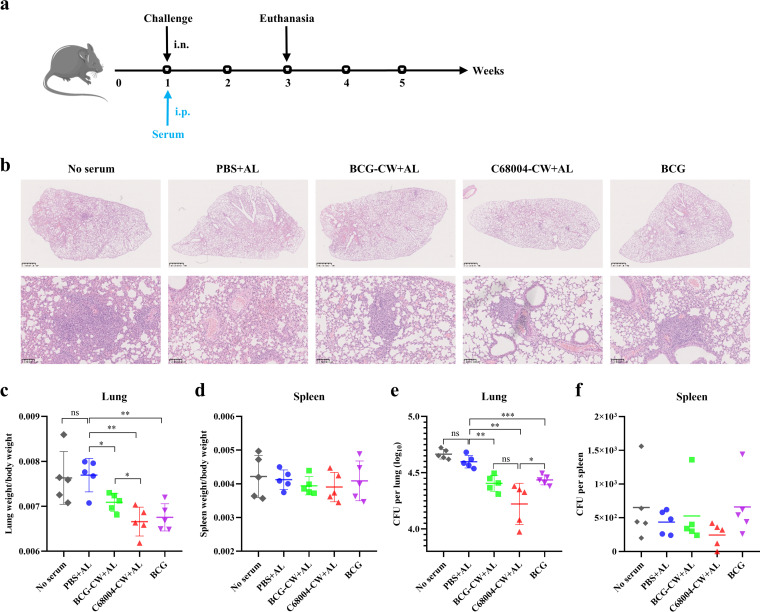
Passive transfer of CW serum prophylactically improves the course of M. bovis infection. (a) Experimental strategies for evaluating CW serum for prevention of M. bovis infection. Five groups of C57BL/6 mice (*n = *5 per group) were injected intraperitoneally (i.p.) with 200 μL of BCG-CW serum, C68004-CW serum, BCG serum, PBS serum, or the negative control, PBS. Immunized mice were infected intranasally (i.n.) with 100 CFU of M. bovis 5 h after immunization with the serum and euthanized to assess the lesions in the lungs 2 weeks after infection. (b) Representative images of lung lobes with hematoxylin and eosin (H&E) staining show histopathological changes in M. bovis-infected mice. Top, scale bars show 1 mm; bottom, scale bars show 100 μm. (c to f) The organ coefficients of the lung (c) and spleen (d) and the bacterial burdens of the lung (e) and spleen (f) were determined 2 weeks postinfection. Data are shown as mean values ± SD and were analyzed with the unpaired two-tailed *t* test. *, *P < *0.05; **, *P < *0.01; ***, *P < *0.001; ns, not significant.

To investigate the protective effect of CW serum against M. bovis *in vivo*, C57BL/6 mice were treated with the immunized-mouse serum via the intraperitoneal (i.p.) route 5 h before being challenged with M. bovis and were sacrificed 2 weeks later to assess the pathological damage to their lungs and spleens (Fig. 4a). Histopathological observation revealed that the lungs of mice treated with CW serum or BCG serum had more confined inflammatory foci and clearer alveolar structure than control mice (Fig. 4b). Additionally, the organ coefficients and bacterial loads of the lungs indicated that CW serum and BCG serum could delay the infection course of M. bovis (Fig. 4c and e). In particular, 2 of 5 mice treated with C68004-CW serum had an approximately 0.6-log reduction in lung CFU compared to the average lung CFU of the control group (Fig. 4e). Furthermore, the average lung CFU of mice treated with C68004-CW serum was lower than that of mice treated with BCG-CW serum, although the lung CFU of the two groups did not show statistical differences (Fig. 4e). As for the spleen, no statistical differences were observed in organ coefficients and bacterial load among groups (Fig. 4d and f). However, it should be pointed out that the average spleen CFU of the C68004-CW serum-treated mice was the lowest, and no bacteria were detected in the spleen of 1 of 5 mice (Fig. 4f). These results indicated that CW serum had preventive activity in M. bovis-infected mice. To further investigate whether the protective effect of CW serum *in vivo* required cellular immunity, we detected the effectiveness of CW serum in athymic (nude) mice, which lack T-cell responses. These results showed that the organ coefficients and bacterial loads of nude mice treated with BCG-CW serum or C68004-CW serum were slightly lower than those of the control group, but not enough to show a statistical difference (Fig. S4), suggesting that the CW serum-mediated protection *in vivo* required an element of functional T-cell activity.

**FIG 5:**
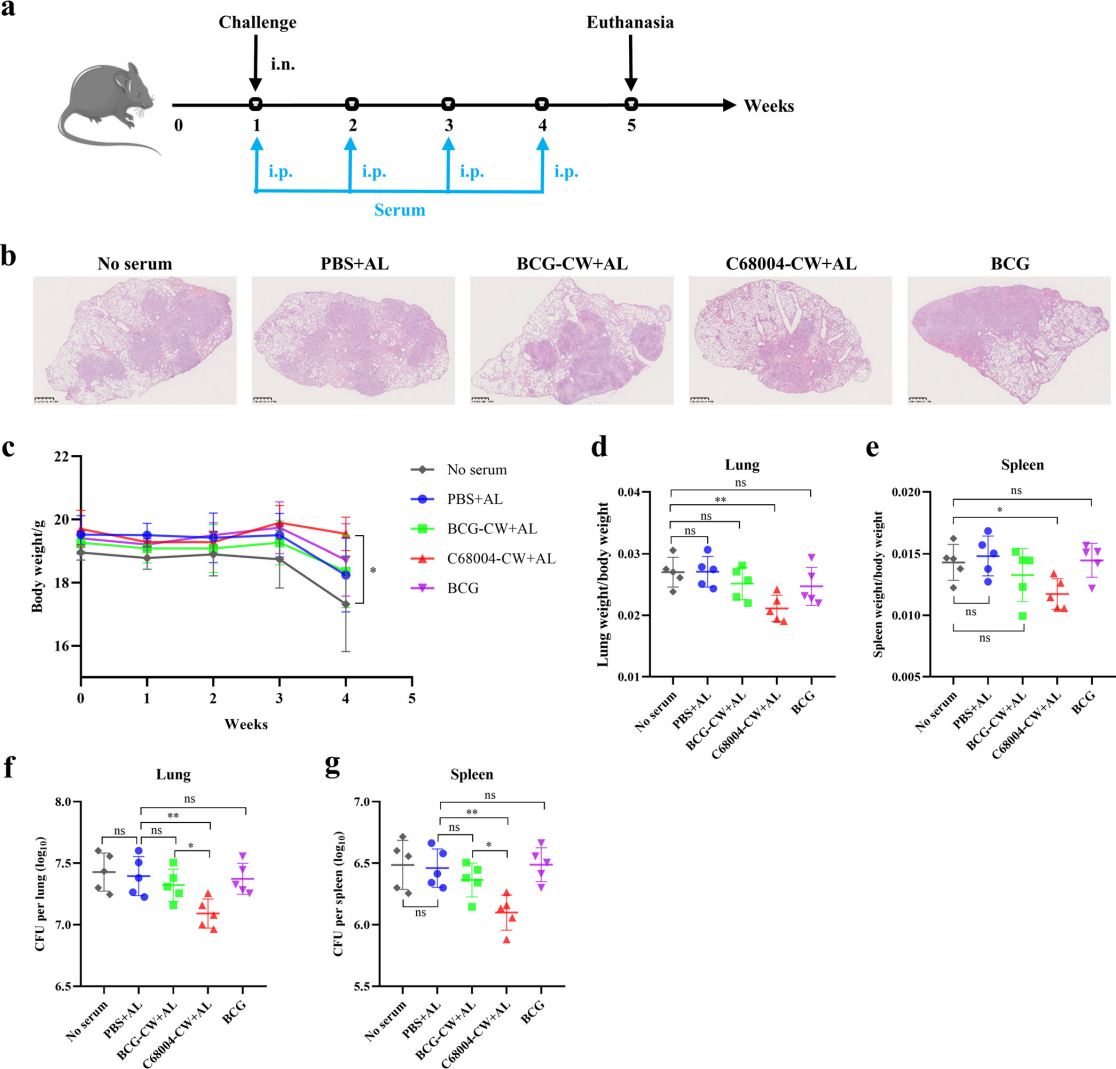
Passive transfer of C68004-CW serum therapeutically modifies the course of M. bovis infection. (a) Experimental design for evaluation of the therapeutic effect of CW serum. C57BL/6 mice were challenged intranasally (i.n.) with 100 CFU of M. bovis. Then, the mice were injected via the intraperitoneal (i.p.) route with 50 μL of BCG-CW serum, C68004-CW serum, BCG serum, PBS serum, or PBS 1 day after infection and then once a week 4 times. The mice were necropsied after the last administration to evaluate the therapeutic effect of serum. (b) Representative images of the H&E-stained lung lobes show histopathological changes in M. bovis-infected mice. Scale bars, 1 mm. (c) During this period of infection, the body weights of M. bovis-infected mice were recorded. (d to g) The organ coefficients of lung (d) and spleen (e) and the numbers of viable bacteria in the lungs (f) and spleen (g) were determined. Data are shown as mean values ± SD and were analyzed with the unpaired two-tailed *t* test. *, *P < *0.05; **, *P < *0.01; ***, *P < *0.001; ns, not significant.

Subsequently, we examined the therapeutic effect of immune serum on M. bovis-infected mice. C57BL/6 mice were delivered 4 consecutive doses of immune serum by i.p. injection from 24 h postchallenge and sacrificed at 4 weeks postinfection (Fig. 5a). Histopathological analysis showed that compared with other groups, C68004-CW-treated mice had significantly fewer inflammatory lung lesions and more intact lung morphology (Fig. 5b). Additionally, the body weight and the organ coefficients of the lungs and spleen indicated that continuous delivery of C68004-CW serum attenuated the degree of pathogenesis of M. bovis in mice (Fig. 5c and e). Similar to other indicators, the bacterial loads in lungs and spleens of mice treated with C68004-CW were also reduced by almost 0.4 log CFU compared with the bacterial loads in the control group (Fig. 5f and g). BCG-CW serum and BCG serum showed no inhibitory activity against M. bovis infection, which was comparable to the control group. These results suggested that C68004-CW serum could therapeutically inhibit M. bovis in mice.

Overall, CW serum can inhibit M. bovis in mice, especially C68004-CW serum, which even has a therapeutic effect on M. bovis-infected mice, and this protective effect *in vivo* is dependent on T cells.

### CW immunization results in different characteristics of BCR repertoires.

**FIG 6:**
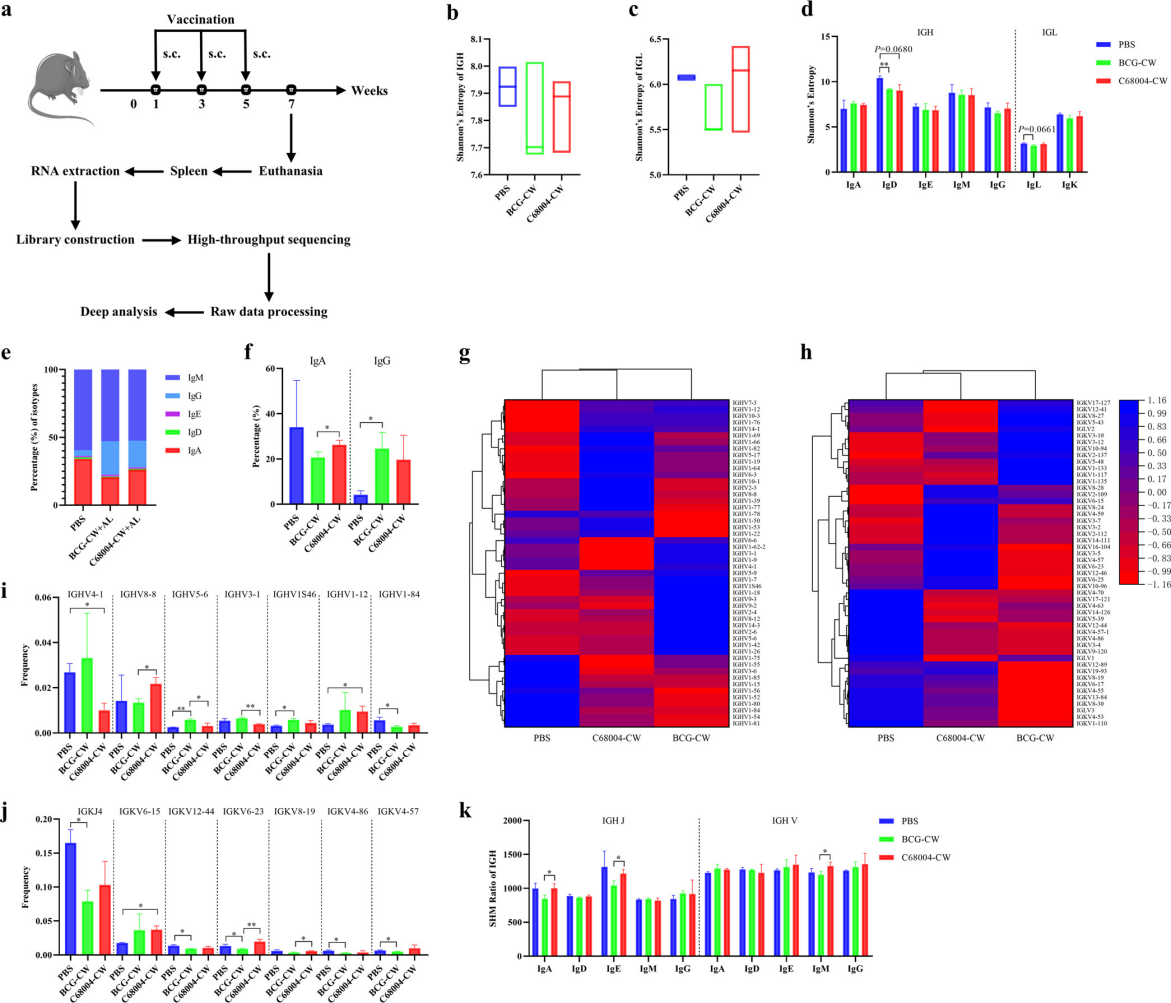
Characterization of BCR repertoire diversity in mice with CW immunization. (a) Experimental strategies for antibody repertoire analysis of CW immunization. C57BL/6 mice in the BCG-CW group (*n *= 3), C68004-CW group (*n *= 3), and PBS group (*n *= 2) were immunized 3 times with BCG-CW, C68004-CW, and PBS, respectively. The immunized mice were subsequently euthanized, and the spleens were collected for RNA extraction, BCR sequencing library construction, and high-throughput sequencing using the Illumina HiSeq platform. The raw data were processed and used for in-depth analysis to generate antibody repertoires. (b to d) Quantification of the repertoire diversities of IGH (b), IGL (c), and their isotypes (d) using Shannon’s entropy. (e) The distribution of 5 isotypes of immunoglobulin. (f) Specific statistical analysis of the changes of IgA and IgG isotypes in each group (details of the data in panel e). (g and h) Gene usages of the V family in IGH (g) and IGL (h). The 50 genes with the highest gene expression frequency in the 3 groups were selected to calculate the average gene expression frequency of each group, and cluster heat map analysis was performed by using OriginPro 2021. (i and j) Specific statistical analysis of V and J family gene usage changes in IGH (i) and IGL (j). (k) SHM ratios of 5 isotypes of immunoglobulin in IGH. Data are shown as mean values ± SD and were analyzed with the unpaired two-tailed *t* test. *, *P < *0.05; **, *P < *0.01.

BCR diversity determines the diversity of antigens recognized by B cells, and the source of BCR diversity lies mainly in V(D)J rearrangements and somatic hypermutation (SHM) of CDR3, etc. (26). To obtain more insight into the functional specificity of the Abs, BCR CDR3 sequences from BCG-CW- and C68004-CW-immunized mice were analyzed to reveal the effects of vaccination on the diversity and immunological characteristics of BCR CDR3 and the relationship between these alterations and the vaccine responses. C57BL/6 mice were euthanized after 3 immunizations, and their spleens were collected for BCR high-throughput sequencing (Fig. 6a). The alignment ratio of all samples was over 60%, indicating that the sequencing results were good enough for data analysis (Table S1).

To quantitatively measure the diversity and complexity of the immune repertoire, Shannon’s entropy is used, with higher entropy values indicating greater diversity (27). Even for different individuals in the same group, the diversity of the immune repertoire also showed relative heterogeneity (Fig. 6b and c). Compared with the PBS group, the mean Shannon’s entropy values of the immunoglobulin heavy chain (IGH) and immunoglobulin light chain (IGL) repertoires in both the BCG-CW and the C68004-CW group were decreased (Fig. 6b and c), indicating that the immunized subjects were dominated by one or a few high-frequency clones. However, these changes lacked statistical significance, likely because of the small sample size or comparison by proportion. Further statistical analysis of isotypes showed that the Shannon’s entropy values of IgD and Ig lambda (IgL) in the BCG-CW group were significantly lower than those in the PBS group, and the difference in values for IgD was statistically significant (Fig. 6d). To better understand the relationship between the repertoires of the 3 groups, Venn diagrams were generated (Fig. S5a and b). Compared with the other groupings, the BCG-CW group and the C68004-CW group shared the fewest CDR3 sequences. Excluding the sequences shared among the three groups, there were 157 and 846 shared IGH and IGL CDR3 sequences between the BCG-CW group and the C68004-CW group (Table S2), respectively, among which the key sequences for the improvement of M. bovis protection might exist.

The length of CDR3 is one of the important determinants of B-cell receptor diversity (28). In the BCG-CW group, C68004-CW group, and PBS group, the lengths of IGH and IGL CDR3 sequences were predominately 12 to 16 amino acids and 11 amino acids, respectively (Fig. S5f and g). However, compared with the other two groups, the distribution of CDR3 lengths in the BCG-CW group tended to be more normally distributed (Fig. S5c and e). Moreover, the BCG-CW group had a significantly higher number of IGH CDR3 sequences with a length of 10 amino acids, but the number of sequences with a CDR3 length of 19 amino acids in the C68004-CW group was also significantly increased, almost 4 times as many as in the BCG-CW group (Fig. S5f). In the distribution of the IGH isotype, the IgG contents of BCG-CW- and C68004-CW-immunized groups were significantly elevated compared with that of the PBS group (Fig. 6e and f), consistent with the previous results showing that BCG-CW and C68004-CW induced high levels of IgG (Fig. 2a and c). Interestingly, the IgA content in the C68004-CW group was remarkably higher than that in the BCG-CW group (Fig. 6e and f). To determine whether CW immunization alters the targeting of V genes, we generated clustered heat maps comparing the use of these genes in IGH and IGL (Fig. 6g and h). The results showed that the gene usage frequencies of IGH and IGL in the V family in the BCG-CW group and the C68004-CW group were different from that in the PBS group, with the latter being relatively more similar to the PBS group. Further statistical analysis of the gene usage frequencies of the V and J families on IGH and IGL revealed that 7 genes each on IGH and IGL were statistically different among the three groups and showed no regular pattern (Fig. 6i and j). For example, compared to the BCG-CW group, the C68004-CW group showed a significantly higher frequency of usage in IGHV8-8, but a lower frequency in IGHV3-1. Additionally, we evaluated SHM, which can introduce new diversity into the repertoire of mature B cells and allow the selection of Abs with high affinity (29). Compared with the BCG-CW group, the C68004-CW group had significantly increased frequencies of IgA and IgE mutations in IGH J genes, as well as IgM mutations in V genes (Fig. 6k). No statistical differences were observed in the mutation frequencies of IGL J and V genes among the three groups (Fig. S5h).

Taken together, CW immunization resulted in different characteristics of CDR3 lengths, isotype distribution, gene usage, SHM, and diversity of BCRs, and further studies need to be done to identify whether these differences are important for protection against M. bovis.

## DISCUSSION

In the current study, we describe our findings that Abs induced by BCG-CW and C68004-CW can enhance FcγR-mediated macrophage phagocytosis of M. bovis, restrict its intracellular growth, and increase phagosome-lysosome fusion. Importantly, the Abs are protective in a mouse model of M. bovis infection, with both preventive and even therapeutic effects, and this efficacy depends on intact T-cell function. Moreover, we also systematically characterize the BCR repertoires, which provides substantial insight into the humoral immune response induced by the CW of a virulent M. bovis strain and that of BCG.

The CW is an ideal anti-TB drug target and can also be used as a vaccine. It had been reported previously that BCG-CW could induce Th1 and Th17 immune responses (18). In our study, compared with BCG-CW, C68004-CW induced more robust cellular immunity (Fig. S3) and stronger humoral immunity, with only the C68004-CW serum showing efficacy in treating M. bovis infection. BCG is an attenuated M. bovis strain mainly lacking the region of difference 1 (RD1) compared to pathogenic M. bovis. The immunodominant antigens ESAT6 and CFP10, often used in anti-TB vaccines, are located in the RD1 region and surface exposed (30, 31). Additionally, it has been reported that the protective efficacy of BCG could be markedly augmented by stable integration of the RD1 region (32, 33). In contrast, strain C68004 remains pathogenic and structurally intact, which may have contributed to its protective efficacy. Therefore, C68004-CW is more suitable for vaccine research than BCG-CW.

In particular, antibodies against LAM and other structural variants of the CW can provide protection against M. tuberculosis infection (8, 10, 34, 35). LAM is an important immunomodulator of M. tuberculosis (36). After shedding from M. tuberculosis, LAM can be recognized and presented by CD1 molecules to activate and recruit T cells to the granuloma (36). Mannosylated LAM (Man-LAM) interacts with mannose receptors on the surfaces of host cells via its mannose cap structure to prevent phagosome maturation, thus promoting the uptake and survival of M. tuberculosis (37). In this study, it was observed that P-L fusion was significantly increased in the presence of C68004-CW serum, possibly because the CW serum blocked the binding of LAM to the mannose receptor. Since oligosaccharides are T-cell-independent antigens, LAM is required to conjugate with proteins to enhance its immunogenicity. It has been reported that AM conjugated with Ag85b could induce high titers of IgG1, IgG2, and IgG3 isotypes of antibodies, instead of only IgG2 (34). Moreover, the AM-Ag85b serum was shown to reduce the transmission capacity of M. tuberculosis by passive administration in mice (34). Currently, LAM-based conjugate vaccines are being used in the development of TB vaccines, with major work focused on exploring oligosaccharide motifs that induce more potent protection of antibodies (38, 39).

Antibody-mediated protection against M. tuberculosis is becoming more and more well explicated, and the effects of Abs in the defense against M. bovis were also preliminarily identified in this study, as follows: (i) Abs promote the uptake of M. bovis by macrophages in a manner dependent on FcRs, (ii) restrict the intracellular growth of bacteria, (iii) increase P-L fusion, and (iv) cooperate with T cells to promote immunity to M. bovis. The nude mouse experiments revealed that the protective effect of CW serum required intact T-cell immunity. T cells may be involved in antibody-mediated M. bovis killing by producing cytokines to activate natural killer (NK) cells and enhance antibody-dependent cytotoxicity responses (6). Early studies reported that gamma interferon (IFN-γ) could be combined with IgA to treat multidrug-resistant TB (MDR-TB) (40). Here, we speculate that the high level of interleukin-17 (IL-17) produced by C68004-CW immunization may play a synergistic role with the anti-CW antibody to inhibit M. bovis infection.

It is time for the widespread use of protective antibody responses in TB vaccine development (6, 23). TB vaccines currently in clinical trials are designed to emphasize cellular immunity, especially Th1 and Th17 immune responses (30, 41, 42). Although clinical vaccines also evoke humoral immunity, they are limited to boosting antibody levels (5), and it is unknown whether these antibody responses confer corresponding protection. An ideal TB vaccine should adopt multiple mechanisms to induce a comprehensive but balanced immune response, with humoral and cellular immunity working together to prevent M. tuberculosis infection (42). The application of “effective humoral immunity” in the TB vaccine may break the bottleneck of TB vaccine development.

Interestingly, the serum obtained by subcutaneous injection of BCG also showed the ability to inhibit M. bovis infection. Both intravenous BCG immunization and mucosal BCG immunization can induce high titers of Abs, which are thought to be associated with the prevention of M. tuberculosis infection (4, 43). In contrast, standard BCG vaccination (intradermal or subcutaneous) only induces lower antibody levels (23). It has been reported that BCG serum from humans could opsonize M. tuberculosis, promoting phagocytosis and inhibiting bacterial intracellular growth (10). Our results show that the serum of C57BL/6 mice immunized subcutaneously with BCG also opsonized M. bovis cells. In a previous passive serum transfer assay, BCG serum did not show any benefit after 4 weeks of challenge with M. tuberculosis (34). However, in our study, BCG serum was protective against M. bovis for at least 2 weeks, which may be related to the spread of bacteria in the early infection period.

From the perspective of the BCR repertoires, at least three reasons may explain the differences in protection between BCG-CW and C68004-CW. First, the C68004-CW group had a higher proportion of long-IGH CDR3 than the BCG-CW group, and the number of sequences with a length of 19 amino acids was almost 4 times that of the latter. The high percentage of a specific CDR3 sequence indicates the amplification of its corresponding cell clones. Long-IGH CDR3 is associated with self-reactive or polyreactive Abs (44), which may be related to the protection. Second, the C68004-CW group showed more antibody affinity maturation than the BCG-CW group. Upon encountering an antigen, BCRs rapidly undergo SHM and class switch recombination (CSR) to improve their binding ability to the antigen and enable affinity maturation (45). Compared with the BCG-CW group, the C68004-CW group had a larger IgA isotype distribution, and the IgA isotype has been shown to protect against M. tuberculosis infection (40, 43, 46–49). Recently, a human monoclonal IgA antibody combined with IFN-γ was shown to significantly inhibit pulmonary MDR M. tuberculosis infection in mice (40, 46). IgA was also identified as a related indicator of local protection in BCG mucosal immunity against M. tuberculosis (43). Additionally, the SHM of the IGH repertoires in IgA, IgE, and IgM were also increased, indicating that they may have undergone affinity maturation to better adapt to antigens. Third, the two groups showed statistical differences in the usage of a total of five genes in the IGH and IGL V family. The diversity of the CDR3 region encoded by the V(D)J gene has the largest theoretical contribution to repertoire diversities, while the V gene is the longest and most variable (26, 50). The preference for gene usage represents the preferential selection of antigens. Nonetheless, these three factors may regulate the protection against M. bovis independently or together, which requires further verification.

Although this study offers insights into the mechanisms underlying the phenotypic heterogeneity of CW immunization, it also has important limitations, including variable immune responses to the same stimulus among individuals and the question of distinguishing antigen-specific changes in the BCR repertoires from control repertoire diversity. In fact, this is the common problem of BCR sequencing in vaccine research (45). The effects may be mitigated to some extent by increasing the sample size and isolating antigen-specific B cells. BCR sequencing provides immunology with a tool to enhance our understanding of humoral immunity and determine the relevance of immune protection after vaccination. Moreover, BCR sequencing can serve as a technical reserve for rapid neutralizing monoclonal antibody discovery. In a recent paper, potent severe acute respiratory syndrome coronavirus 2 (SARS-COV-2) neutralizing Abs were quickly and effectively identified by high-throughput single-cell BCR sequencing (51, 52). The next step in our plan is to screen for effective anti-TB vaccines from the obtained BCR repertoires.

Although the protection of CW serum against M. bovis is moderate, it is critical to note that the protective effect of CW serum in mouse infection models and *in vitro* was assessed with M. bovis cultured using detergent, where some epitopes were reduced (24). Consequently, the protective ability of CW serum obtained using encapsulated bacteria would be elevated. The M. bovis genome is 99.9% or more homologous to the M. tuberculosis genome (53), which means that its CW can be considered for the development of new TB vaccines. In summary, our findings demonstrate that CW-targeting Abs can induce protection against virulent M. bovis, and we characterize the BCR repertoires of protective Abs for the first time, providing new insights for developing new preventive or therapeutic TB vaccines.

## MATERIALS AND METHODS

### Ethics statement.

All animal studies were carried out in strict accordance with the guidelines of the Chinese Association for Laboratory Animal Sciences and approved by The Laboratory Animal Ethical Committee of China Agricultural University (20110611-01).

### Bacterial culture, infection, and CW fraction preparation.

M. bovis BCG strain Pasteur 1173P2 and M. bovis strain C68004 (21) were grown in Difco Middlebrook 7H9 broth (SKU 271310; Becton, Dickinson and Company) supplemented with 10% (vol/vol) oleic acid-albumin-dextrose-catalase (OADC) (SKU 212351; Becton, Dickinson and Company) and sodium pyruvate (DE-0342A; Biodee) at 4 mg/mL and with or without 0.05% (vol/vol) Tween 80 (P1754; Sigma) for 2 to 3 weeks at 37°C. In this study, the bacteria used to infect animals or cells were cultured with Tween 80 to make infection more accurate, and the bacteria used in other experiments were cultured without Tween 80 unless otherwise specified.

BCG or M. bovis C68004 was grown to an optimal density (an optical density at 600 nm [OD_600_] of 0.4), collected by centrifuging at 4,000 rpm for 10 min, and washed 3 times with PBS. Then, the pellet was suspended in PBS, passed through a 25-gauge-needle syringe 10 times, and centrifuged at 300 × *g* for 5 min. The supernatant was measured by the OD_600_ to assess bacterial concentration, diluted to the target concentration, and ultimately used to infect cells or animals.

The cell wall fraction was prepared as previously described (9, 54). BCG or M. bovis C68004 was grown in culture medium as described above without Tween 80 and washed 3 times with PBS at room temperature. The pellet was suspended in PBS and lysed by sonication on an ice bath. This sonicate was centrifuged at 3,000 × *g* for 5 min at 4°C, and then the supernatant was centrifuged at 27,000 × *g* for 1 h at 4°C. The pellet from this centrifugation, representing the CW fraction, was suspended in PBS.

### Immunofluorescence assay.

The immunofluorescence assay was performed as previously described (8). M. bovis, grown in the presence and absence of 0.05% Tween 80, was fixed with 2% paraformaldehyde (PFA) at 4°C for 1 h and then placed on poly-l-lysine slides (Citotest) and blocked with Immunol staining blocking buffer (product number P0102; Beyotime Biotechnology) for 1 h at room temperature. Murine serum samples (1:100) were added and incubated with M. bovis at 4°C overnight. After rinsing in PBS, the secondary antibody, fluorescein isothiocyanate (FITC)-labeled goat anti-mouse IgG (1:500; Solarbio), was added for 1.5 h at room temperature in the dark. After washing, the slides were mounted with antifade mounting medium (product number P0126; Beyotime Biotechnology) and viewed using a Zeiss Observer microscope.

### Immunization and infection.

Six- to 8-week-old female C57BL/6 mice were purchased from SPF (Beijing) Biotechnology Co., Ltd., randomly divided into 4 groups, and maintained in biosafety level 3 (ABSL-3) animal facilities of China Agricultural University. Animals were immunized subcutaneously (s.c.) 3 times with 50 μg of BCG-CW or C68004-CW, including 1% (wt/vol) alum. Negative-control mice received 3 s.c. injections of PBS, including 1% (wt/vol) alum, while positive-control mice were vaccinated s.c. once with 1 × 10^6^ CFU BCG. Immunizations were performed every 2 weeks. Immunized mice were infected intranasally (i.n.) with 100 CFU of virulent M. bovis 4 weeks after the last immunization or 6 weeks after BCG immunization. After 24 h following infection, 3 infected mice were euthanized for evaluation of the actual infection dose delivered to the lungs. The tissues were homogenized in PBS plus 0.05% Tween 80, serially diluted, and plated on Middlebrook 7H10 agar supplemented with 10% OADC to determine CFU. The actual infection dose delivered to the lungs was verified as between 100 and 200 CFU/mouse in all animal experiments. Mice were sacrificed 4 weeks after the challenge. The organ coefficient (ratio of organ weight to the total body weight) and the CFU of lungs and spleens were calculated and quantified.

### ELISA.

Enzyme-linked immunosorbent assays (ELISAs) were performed as previously described (7). Briefly, ELISA plates were coated with 100 μL of BCG or M. bovis cell wall fractions (5 μg/mL) in 0.05 M carbonate bicarbonate (CBC) buffer (pH 9.6) overnight at 4°C. The next day, the plates were washed 3 times with PBS containing 0.05% Tween 20 (PBST) and blocked with 5% nonfat milk powder in PBS (blocking buffer) for 2 h at 37°C. Then, the plates were incubated with serum samples from immunized mice (1:1,000) for 1 h at 37°C. Next, the plates were washed and incubated with horseradish peroxidase (HRP)-conjugated goat anti-mouse IgG, IgG1, IgG2a, or IgG3 (1:10,000; Abcam) for 1 h at 37°C. After washing again, the plates were developed with tetramethylbenzidine (TMB) for 15 min at room temperature. Finally, the reaction was stopped by 0.1 N sulfuric acid and the optical densities were measured at 450 nm.

For whole-bacterial-cell ELISA, M. bovis cells grown in the culture medium without Tween 80 were killed by heating to 80°C for 2 h. Bacterial cells were suspended with PBS, dispersed by drawing up and expelling the bacterial suspension 10 times through a 25-gauge-needle syringe, and then adjusted to a concentration of 1 × 10^8^ CFU/mL. ELISA plates were coated with 50 μL of the bacterial cells, dried at 56°C, and incubated with −20°C precooled methanol for 15 min at room temperature. After an additional washing step, the plates were incubated with blocking buffer and processed as indicated above.

### Phagocytosis by flow cytometry.

The phagocytosis assay was carried out as previously described (55). When grown to log phase, BCG was collected by centrifuging at 4,000 rpm for 10 min. To conjugate with FITC, the pellet was stained by FITC (1 mg/mL) in 0.1 M CBC buffer for 2 h on a shaker at 37°C and then washed 3 times with PBS to remove the unbound FITC. J774A.1 macrophages were infected with FITC-conjugated BCG at a multiplicity of infection (MOI) of 20:1 or 5:1 and coincubated with 10% heat-inactivated serum for 3 h or 24 h at 37°C with 5% CO_2_. After washing, the macrophage cells were resuspended in 500 μL of 4% PFA and then measured using the BD Fortessa cytometer. Data were analyzed using FlowJo software.

### Mycobacterial intracellular growth inhibition assay.

The mycobacterial intracellular growth inhibition assay was carried out as previously described (10). J774A.1 cells were infected with M. bovis at an MOI of 10:1 and coincubated with Dulbecco modified Eagle medium (DMEM) containing 10% heat-inactivated serum at 37°C with 5% CO2, followed by washing to remove extracellular bacteria. After washing, 10% heat-inactivated serum was added, and this time point was counted as 0 h postinfection. The cells were lysed with 0.1% Triton X-100 at 0 h, 24 h, and 48 h postinfection. Serial dilutions of the lysate were plated on Middlebrook 7H10 agar plates supplemented with 10% OADC, the plates were incubated at 37°C for 2 to 3 weeks before counting CFU, and finally, the average CFU from triplicate wells were calculated.

The depletion or blocking experiments were performed as previously described (7, 8). To deplete Abs against CW, heat-inactivated serum was diluted with DMEM and then incubated with a CW pellet in a shaker at 4°C overnight. After centrifugation, the supernatants corresponding to the antibody-depleted-serum dilution were collected. This depletion process was performed 3 times before the depleted serum was passed through a 0.22-μm Spin-X filter. To block FcγR, 10 μg/mL anti-mouse CD16/CD32 (70-AM016-100; Multisciences) or rat IgG2b isotype control (70-CRG2b05-10; Multisciences) antibody was added to the cells.

### Assessment of phagosome-lysosome fusion.

J774A.1 macrophages were grown on coverslips in 24-well cell culture plates for 12 to 16 h. Then, the cells incubated with 10% heat-inactivated serum were infected with FITC-conjugated BCG at an MOI of 20:1 for 1 h, washed 3 times with DMEM, and incubated with DMEM supplemented with 10% fetal bovine serum (FBS) and 50 nM LysoTracker red DND-99 (product number C1046; Beyotime Biotechnology) for 1 h. After a washing step, the cells were fixed with 4% PFA for 30 min at room temperature. Then, the washed coverslips were mounted on glass microscopy slides with antifade mounting medium (product number P0126; Beyotime Biotechnology). To assess the amount of phagosome-lysosome fusion, approximately 10 to 15 unique images were captured at random under a magnification of ×100 (oil immersion lens) using a Nikon A1HD25 confocal microscope. BCG phagosomes were counted to quantify the percentage of phagosomes that colocalized by means of the LysoTracker staining.

### Passive serum transfer experiments.

C57BL/6 and nude BALB/c (BALB/c nu/nu) female mice between 6 and 8 weeks old were purchased from SPF (Beijing) Biotechnology Co., Ltd., and maintained under ABSL-3 conditions. After a week of adaptive feeding, C57BL/6 mice were randomly divided into 2 groups: the prevention group and the treatment group.

The prevention group was further caged in groups of 5, and the experiments were carried out as previously described (7, 34). Four groups of mice were injected intraperitoneally (i.p.) with 200 μL of the corresponding serum, which was collected from C57BL/6 mice previously immunized with BCG-CW, C68004-CW, PBS, and BCG. Additionally, a fifth group was injected with PBS as a control. Immunized mice were infected i.n. with 100 CFU of M. bovis 5 h after immunization with the serum and euthanized to assess the lesions in the lungs 2 weeks after infection.

Similarly, the treatment group was divided into the same 5 groups. The difference was that the mice were injected via the i.p. route with 50 μL of corresponding serum or PBS 1 day after infection and then once a week 4 times. The mice were necropsied to evaluate the therapeutic effect of serum after the last administration.

As for nude mice, the experiment was carried out as described above for the prevention group, but the mice were divided into three groups, namely, a BCG-CW serum group, a C68004-CW serum group, and a PBS control group.

### Histopathological analysis.

The same lobe of lung tissue from different mice was fixed in 10% formalin solution, embedded in paraffin, and cut into 3-μm-thick tissue sections. Thens the sections were mounted on glass slides, deparaffinized, and stained with hematoxylin and eosin (H&E) (G1120; Solarbio). The slides were scanned with the Hamamatsu NanoZoomer-S60 digital slide scanner.

### BCR deep sequencing.

B-cell receptor (BCR) deep sequencing was carried out by the Wuhan SeqHealth Tech Co., Ltd. (http://seqhealth.cn/list/2.html), as previously described (50). Briefly, the library was constructed using the KC-Digital reactive BCR-SeQ library prep kit for Illumina (DT0811-02; SeqHealth), combined with unique identifiers (UIDs). Then, the amplified PCR products were sequenced by using the Illumina HiSeq platform, and the raw sequencing data were subjected to quality control via SOAPnuke software (version 2.1.0) (56). Through the internal software of the sequencing company, similar reads under the same UIDs were merged for error correction and removal of duplicate reads. Next, the resulting FASTA sequences were mapped to the V, D, and J gene fragments from the IMGT database (57) by using MiXCR software (version 3.0.3) (58) to acquire the genetic information, rearrangement pattern, and CDR3 sequences of the reads.

### Statistical analysis.

*R*^2^ values represent the Gauss distribution fitting values and were calculated using the CDR3 length statistics file from the OriginPro 2021 analysis. Shannon’s entropy (27) was used to estimate BCR clonal diversity, calculated by summing the frequency of each clone times the log (base 2) of the productive reads with the same frequency overall in a sample.

All cell assays were performed on three separate occasions, and the data are expressed as mean values ± standard deviations (SD). Parametric data were analyzed by using the unpaired two-tailed *t* test with a significance threshold of a *P* value of <0.05. Statistical analysis was performed in GraphPad Prism (version 7.0) and OriginPro 2021 (version 9.8.0.200). Venn diagrams were plotted from the exported data using the R package VennDiagram.

### Data availability.

All relevant data are within the manuscript and its supplemental material.
