# Supplementary material for: Antibodies Targeting the Cell Wall Induce Protection against Virulent Mycobacterium bovis Infection
Source: Microbiol Spectr. 2023 Feb 27;11(2):e03431-22. doi: 10.1128/spectrum.03431-22 (PMC10100962; doi:10.1128/spectrum.03431-22)
Supplement: Supplemental file 1 — Fig. S1 to S5. Download spectrum.03431-22-s0001.pdf, PDF file, 0.7 MB [file spectrum.03431-22-s0001.pdf]

Fig S1

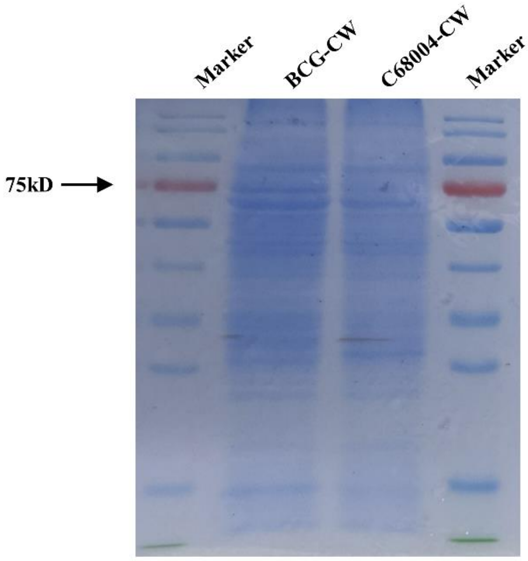

Isolation of CW. Protein components in cell wall fractions were subjected to SDS-PAGE and protein bands were visualized by Coomassie blue staining.

Fig S2

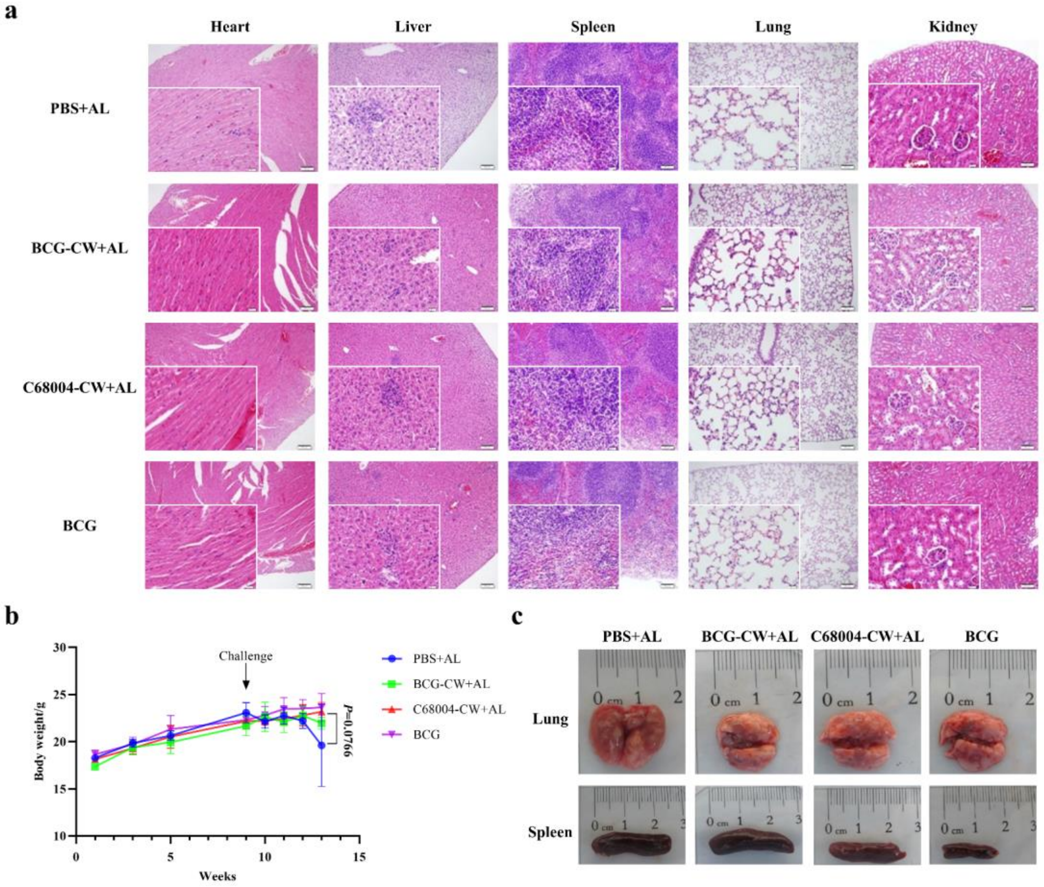

Clinical parameters in the CW-induced mouse protection assay. (a) To assess safety and toxicity of CW vaccinations, histopathological evaluation of organs in mice after the last vaccination but before challenge was performed with H&E staining. Scale bar: 100 μm and 20 μm. (b) During the experimental period, the body weight of mice was recorded. (c) The representative images of lung and spleen showed the gross pathological changes of *M. bovis*-infected mice. Data were analyzed with an unpaired two-tailed t-test.

Fig S3

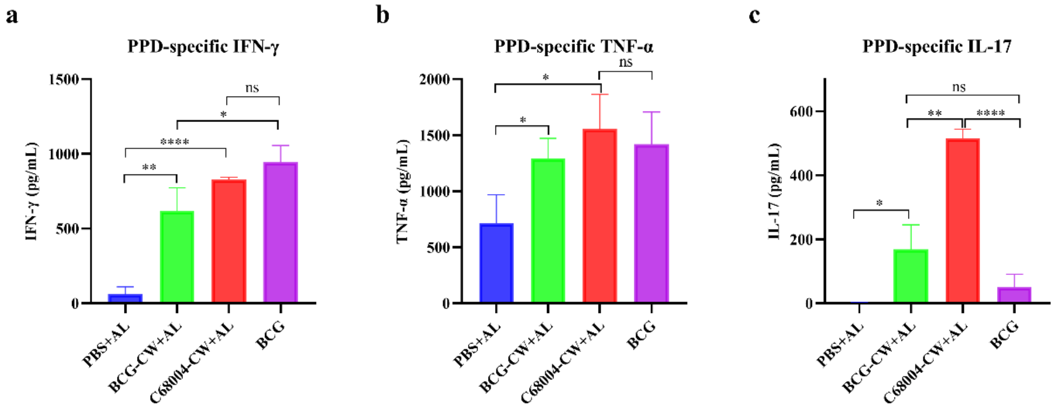

CW contribute to produce specific Th1 and Th17 cytokines. Two weeks after the last immunization, splenocytes were isolated from BCG-CW-, C68004-CW-, PBS-, BCG-immunized mice (n = 3), stimulated with PPD for 12 h, and then concentrations of IFN-γ (a), TNF-α (b), and IL-17 (c) in cell supernatants was determined by ELISA. Data were shown as means ± SD, and analyzed with an unpaired two-tailed t-test. *$P < 0.05$; **$P < 0.01$; ***$P < 0.001$; ****$P < 0.0001$; ns, not significant.

Fig S4

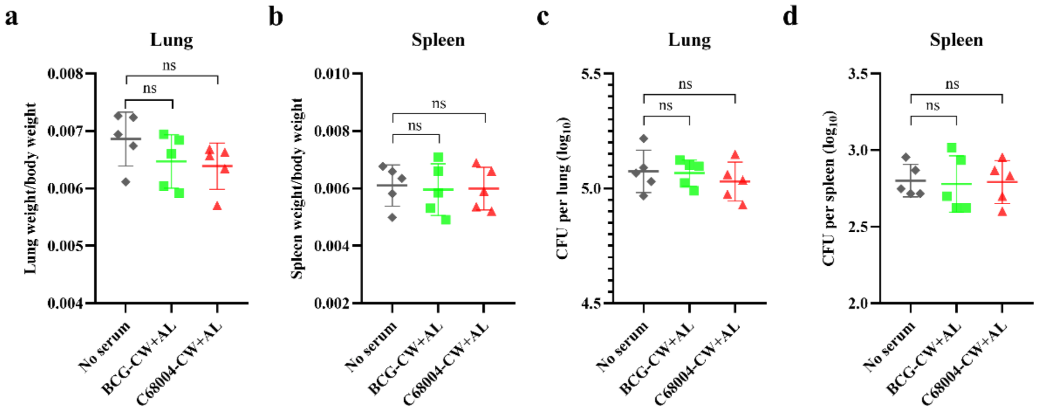

Protective antibody requires intact T-cell immunity in mice. 3 groups of BALB/c nu/nu mice (*n* = 5 per group) were injected intraperitoneally (i.p) with 200 μl of BCG-CW serum, C68004-CW serum, as well as the negative control PBS, respectively. Immunized mice were infected intranasally (i.t.) with 100 CFU of *M. bovis* 5 h after immunization with the serum, and euthanized to assess the lesions in the lungs 2 weeks after infection. (a-d) The organ coefficient of lung (a) and spleen (b), the number of viable bacteria in the lung (c) and spleen (d) were determined 2 weeks post infection. Data were shown as means ± SD, and analyzed with an unpaired two-tailed t-test. Ns, not significant.

Fig S5

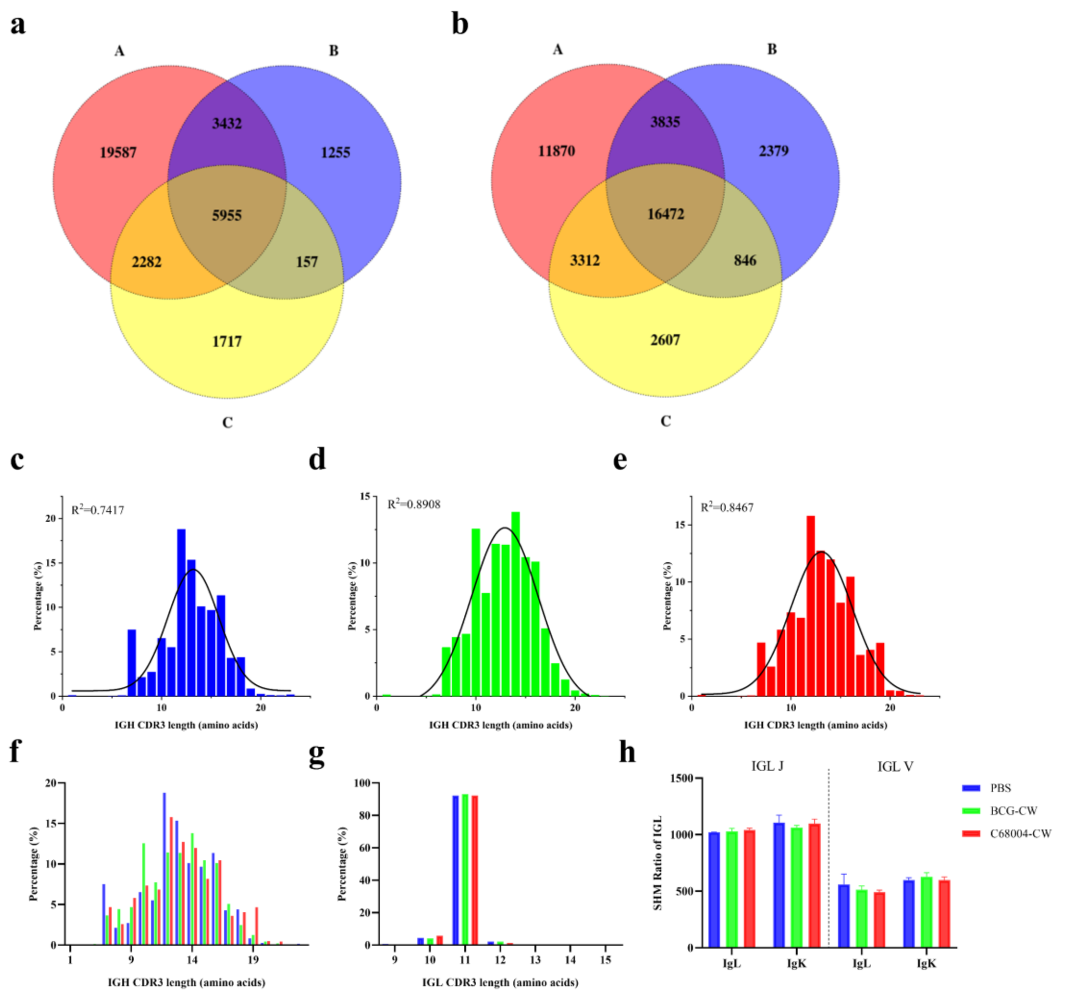

The analysis of the BCR repertoires. (a and b) Venn diagram showing the number of shared clones of IGH (a) and IGL (b) CDR3 among PBS (A, *n* = 2), BCG-CW (B, *n* = 3) and C68004-CW (C, *n* = 3) groups. (c-e) To compare the CDR3 length among PBS, BCG-CW and C68004-CW groups, the CDR3 length with frequency more than 0.01% was collected, and the average of CDR3 length in each group was calculated. IGH CDR3 length distribution of PBS (a), BCG-CW (b) and C68004-CW (c) groups. R^2 represented Gauss distribution fitting value, and the closer the value was to 1, the closer the distribution was to the normal distribution. (f-g) Comparison of IGH (f) and IGL (g) CDR3 length distribution among 3 groups. (h) The SHM ratio of IgL and IgK class among 3 groups

were performed. Data were shown as means or means ± SD.
